# Fit to Forgive: Effect of Mode of Exercise on Capacity to Override Grudges and Forgiveness

**DOI:** 10.3389/fpsyg.2017.00538

**Published:** 2017-05-08

**Authors:** C. Ward Struthers, Elizabeth van Monsjou, Mariam Ayoub, Joshua R. Guilfoyle

**Affiliations:** Department of Psychology, York University, TorontoON, Canada

**Keywords:** forgiveness, grudges, exercise, self-control, self-regulation

## Abstract

Forgiveness is important for repairing relationships that have been damaged by transgressions. In this research we explored the notion that the mode of physical exercise that victims of transgressions engage in and their capacity to override grudges are important in the process of forgiveness. Two exploratory studies that varied in samples (community non-student adults, undergraduate students) and research methods (non-experimental, experimental) were used to test these predictions. Findings showed that, compared to anaerobic or no exercise, aerobic and flexibility exercise facilitated self-control over grudges and forgiveness (Studies 1 and 2), and self-control over grudges explained the relation between exercise and forgiveness (Study 2). Possible mechanisms for future research are discussed.

## Introduction

The physical and psychological benefits of exercising are well documented but less is known about how it may benefit relationships. Research examining the physical benefits of exercise shows that those who engage in regular exercise have improved cardiovascular functioning (Zoeller, 2007), reduced obesity-related health problems such as diabetes (Corsica and Perry, 2003), increased cerebral blood flow (Scheinberg et al., 1954; Thomas et al., 1987; Seifert and Secher, 2011; Paillard, 2015), and improved cognitive functioning (Diamond et al., 2007; Chang et al., 2012a,b; Barnes, 2015; Chang and Etnier, 2015). Even more impressive is research suggesting that exercise facilitates structural brain changes (Voelcker-Rehage and Niemann, 2013) and the generation of new brain cells (Cotman et al., 2007), reduces risk of dementia (Larson et al., 2006; Barnes, 2015) and some forms of cancer (Batty and Thune, 2000; Friedenreich et al., 2001; Gilliland et al., 2001; Van der Kooy et al., 2001), and increases longevity (Kokkinos et al., 2007). Like the physical benefits, research on exercise’s psychological benefits has demonstrated that individuals who exercise regularly experience less stress, depression, and anxiety (Dunn et al., 2001; Singh et al., 2001; Castro et al., 2002) and improved mood, self-esteem, and self-control (Sonstroem, 1997; Oaten and Cheng, 2006; Schmidt et al., 2016; Smith, 2006). However, despite these growing bodies of evidence regarding the virtues of exercise on physical and psychological functioning, much less is known about the potential benefits of exercise on functioning in relationships, particularly following a transgression. The purpose of this research was to address this gap by exploring the association between individuals who engage in specific types of exercise and forgiveness, which is an important interpersonal factor in the repair of relationships following a transgression.

Social bonds are crucial for the psychological well-being and survival of human beings (Bowlby, 1969; Buss, 1990; Baumeister and Leary, 1995; Berscheid and Reis, 1998; Myers, 2000; Ryan and Deci, 2000; Tooby et al., 2006). However, in the process of developing and maintaining relationships, individuals often commit transgressions that can jeopardize these bonds. Fortunately, people have developed various ways to repair the damage caused when one person hurts another. One of these ways is to forgive transgressors (McCullough, 2008). Based on several definitions of forgiveness in which victims transform unfavorable evaluations and behaviors into more favorable evaluations and behaviors that benefit the transgressor (see Exline and Baumeister, 2000; McCullough, 2001; Struthers et al., 2008; Carlisle et al., 2012; Dorn et al., 2013; Worthington et al., 2015), we defined forgiveness as a motivated decision to transform unfavorable evaluations and antisocial behavior toward a transgressor into more favorable evaluations and prosocial behavior.

Most germane to the purpose of this research is the empirical literature that has linked forgiveness to physiological functioning such as efficient use of glucose (DeWall et al., 2010), psychological functioning such as greater self-control over grudges (Finkel and Campbell, 2001; Burnette et al., 2014), social functioning such as reduced antisocial interactions (Aquino and Douglas, 2003; Exline et al., 2004) and improvements in prosocial interactions (Karremans and Van Lange, 2004; Karremans et al., 2005), as well as improvements in functioning of relationships (Rye and Pargament, 2002; Fincham et al., 2004; Hoyt et al., 2005; Paleari et al., 2005; Braithwaite et al., 2011; Ysseldyk and Wohl, 2012; Kato, 2016). With few exceptions (e.g., Luchies et al., 2010), this research shows that forgiveness has numerous benefits for relationships. For instance, forgiving in romantic relationships is linked to less relationship dissolution (Kato, 2016), greater relationship commitment (Ysseldyk and Wohl, 2012), increased relationship satisfaction (Braithwaite et al., 2011), and better conflict resolution (Fincham et al., 2004).

Despite the potential benefits of forgiveness in repairing relationships, it is a difficult process to initiate and carry out because victims must first override the inclination to protect themselves from future transgressions by harboring grudges (Heider, 1958; Baumeister et al., 1998; Exline and Baumeister, 2000; McCullough, 2008; McCullough et al., 2013; Burnette et al., 2014). We define a grudge as hanging on to negative sentiment and judgments toward transgressors by ruminating or repetitively thinking about the transgression. Research shows that ruminating about a transgression increases negative feelings about the transgressor and deters prosocial responses such as forgiveness (Rusting and Nolen-Hoeksema, 1998; Witvliet et al., 2001; McCullough et al., 2007; Ray et al., 2008). Although grudges provide short-term benefits, such as promoting vigilance against future transgressions (Rapske et al., 2010), over the long-term, persistent grudges are likely to prevent victims from forgiving because they impair victims’ ability to reappraise the negative event, their capacity to diminish their negative sentiment, and hinder reconciliation (McCullough et al., 2007; Denson et al., 2011).

In the following research, we tested the idea that different types of exercise influence victims’ capacity to override grudges, which then facilitates forgiveness (Burnette et al., 2014). The capacity to override a desired response such as harboring a grudge, is referred to as self-control, and has been shown to be important in the regulation and maintenance of relationships (Tangney et al., 2004; Baumeister et al., 2005). Despite ongoing debate in the literature on self-control regarding the mechanisms that explain the operation of self-control (Hagger et al., 2010; Baumeister, 2014; Carter and McCullough, 2014; Inzlicht et al., 2014; Baumeister and Vohs, 2016; Beurms and Miller, 2016; Cunningham and Baumeister, 2016; Evans et al., 2016), its study has blossomed over the past two decades. By exercising self-control, people are able to resist the temptation to act in self-serving ways, and, instead, act in socially adaptive ways, which facilitates pleasant and long lasting relationships with others (Baumeister et al., 2005). We propose that the self-control required to override grudges is influenced by cognitive control processes, which are differentially influenced by exercise (Colcombe and Kramer, 2003; Smith et al., 2010; Guiney and Machado, 2013; Barnes, 2015; Chang and Etnier, 2015). We further argue that the self-control needed to override a grudge exerts downstream effects on victims’ capacity to forgive (Burnette et al., 2014). Although research shows that exercise is linked to the ability to regulate negative emotions such as the ones involved in harboring a grudge, little is known about the role that different types of exercise may play in the process of overcoming negative emotions (Antúnez et al., 2013).

There are three main categories of physical exercise: anaerobic, aerobic, and stretching (Johnson, 1998). *Anaerobic* exercise is defined by short bursts of extremely high energy, such as lifting weights, that limits cardiovascular functioning (Levchuck et al., 2000). *Aerobic* exercise, such as jogging and aerobics classes, is characterized by intense and sustained activity that stimulates and strengthens the heart and lungs and improves cardiovascular functioning (Taylor and Sirois, 2009). *Stretching* is the process of stationing body parts in positions that increase flexibility and lengthen ligaments, tendons, and skin, for instance, stretching classes (McAtee and Charland, 2007). Stretching can also positively influence cardiovascular functioning (Paillard, 2015; Zheng et al., 2015).

Our research is exploratory and, in part, based on the idea that different modalities of exercise might influence the operation of self-control over grudges through improved cognitive control. Cognitive control is a set of cognitive processes including inhibitory control, attentional control, working memory, and cognitive flexibility (Diamond, 2006). Because the process of exercising self-control involves inhibiting dominant responses, such as harboring grudges, to allow other more appropriate responses, such as forgiveness, it is most directly linked to inhibitory cognitive control processes (Robinson et al., 2010; Inzlicht et al., 2014). We further argue that the self-control exerted when overriding grudges has important downstream implications for the interpersonal process of forgiveness, such that those who effectively override grudges are more forgiving.

We propose that different types of exercise affect cardiorespiratory functioning (Mazzeo et al., 1998; Seifert and Secher, 2011), cognitive control (Hillman et al., 2008; Pontifex et al., 2011; Erickson et al., 2014), and self-control (Audiffren and Andre, 2015; Barnes, 2015). Because aerobic and stretching exercises are characterized by efficient cardiorespiratory functioning (e.g., Mazzeo et al., 1998; Seifert and Secher, 2011; Zheng et al., 2015) and improved cognitive control (e.g.,Voss et al., 2010; Erickson et al., 2015), we expect that engaging in these types of exercise should enable people to exert greater self-control over grudges (Pontifex et al., 2011; Burnette et al., 2014; Erickson et al., 2014; Audiffren and Andre, 2015). In comparison, because anaerobic exercise is defined by short bursts of extremely high energy that limits cardiorespiratory functioning (Jung et al., 2012; Lefferts et al., 2014) and cognitive control (e.g., Chang et al., 2012b), we propose that engaging in this form of exercise should limit self-control over grudges (Oaten and Cheng, 2006; Pontifex et al., 2011). Moreover, we predicted that one downstream gain for victims of transgressions who engage in aerobic and stretching exercises would be greater forgiveness than those who engage in anaerobic exercises.

### Overview of Current Research

A growing body of research shows that physical exercise is associated with improved cognitive control implicated in the operation of self-control (e.g., Colcombe and Kramer, 2003; Hillman et al., 2008; Davranche and McMorris, 2009; Smith et al., 2010; Pontifex et al., 2011; Chang et al., 2012b; Guiney and Machado, 2013; Gomez-Pinilla and Hillman, 2013; Hung et al., 2013; Audiffren and Andre, 2015). This research has also begun to focus on the modality of exercise and cognitive functioning (Colcombe and Kramer, 2003; Budde et al., 2008; Smith et al., 2010; Chang et al., 2012b; Harveson et al., 2016), suggesting that type of exercise might be important in the operation of self-control over grudges. Research also shows a link between exercise and improved self-control in the long-term (Oaten and Cheng, 2006; Audiffren and Andre, 2015). Moreover, an independent association between cognitive control and self-control has been found (Hagger et al., 2010). Finally, self-control is negatively associated with harboring grudges and positively linked to forgiveness (Burnette et al., 2014).

Despite the independent associations between exercise and self-control, and self-control and forgiveness, researchers still do not know if, and how, type of exercise, self-control over grudges, and forgiveness interrelate. Thus, the primary aim of this research was to explore the association between exercise, self-control over grudges, and forgiveness. We predicted that aerobic and flexibility (i.e., stretching) exercise would decrease grudge holding and increase forgiveness compared to anaerobic exercise. In addition, we also wanted to explore the mediational role of self-control over grudges in explaining why the different modes of exercise influence forgiveness. In Study 1 we set out to explore these associations in a community sample of non-student adults and a real-life transgression occurring within a romantic relationship. In Study 2 we attempted to extend these findings by using a sample of undergraduates and an experimental design (i.e., experimental field). In addition, we tested the mediating role of grudges in explaining why different types of exercise facilitated or hindered the forgiveness process.

## Study 1

In Study 1 we tested the association between exercise and forgiveness by assessing the routine exercise behavior of a community sample of adults, using a retrospective recall of an actual relationship transgression involving a romantic partner, and assessing their forgiveness of their partner.

### Design

A one-way non-experimental design was used to test the association between exercise type (control, anaerobic, aerobic, and stretching) and forgiveness.

### Method

#### Participants

The participants were a community sample of 105 adults who were on average 29.13 years old, *SD* = 11.25, (Male = 53, Female = 51, one did not report gender). Our sample size was determined using a power analysis with four groups, α = 0.05, medium to strong effect size, and 80% power. Participants reported routinely exercising weekly or several times per week and on average rated their current level of fitness as 3.90 (*SD* = 1.49) whereby 1 = *poor/out of shape* and 7 = *excellent/in best shape*. As well, the participants reported that the transgression had occurred within months, *M* = 3.40, *SD* = 0.88, (1 = *days*, 2 = *weeks*, 3 = *months*, 4 = *years*); that they had been in the relationship for an average of 1–2 years, *M* = 3.08, *SD* = 1.15 (1 = *weeks*, 2 = *months*, 3 = *1–2 years*, 4 = *2–3 years*, 5 = *more than 3 years*); and that they were committed to the relationship, *M* = 5.66, *SD* = 1.56, (1 = *not at all committed*, 7 = *very committed*). As compensation, those who completed the study had their names entered into a draw for $100.

#### Materials

##### Exercise behavior

As part of a larger questionnaire, participants indicated the form of exercise they engage in most frequently, including anaerobic, aerobic, stretching, and no exercise (control). Participants were asked, *When exercising I mostly do (choose the one that best fits)*: aerobics (running, jogging, cycling, and swimming); anaerobics (lift weights); stretching (yoga, pilates); or I don’t exercise.

##### Transgression stimulus

Participants were instructed to think about a current or past romantic relationship in which their partner committed a transgression against them. They were then instructed to write about what happened and how it made them feel.

##### Forgiveness

Participants were asked to indicate the extent to which they forgave their partner, 1 = *I would never forgive him/her*, 2 = *I may forgive him/her*, 3 = *I am trying to forgive him/her*, 4 = *I forgave him/her*. Most definitions of forgiveness describe it as a process that takes place over time and therefore we opted for this scale because it reflected our goal to assess where our participants were in terms of the different stages of the forgiveness process ranging from never forgiving to forgiveness. Other formal scales of forgiveness (e.g., the forgiveness scale, Eaton and Struthers, 2006; TRIM, McCullough et al., 1998) tend to assess the extent to which participants would forgive or have forgiven rather than the broader range of stages. Previous research shows that the measure we used in this study correlated with other multi-item formal measures of forgiveness (Struthers et al., 2010). We also assessed the extent to which the participants were still involved in the relationship. Participants were asked if they were still involved in the relationship, 1 = *no my partner and I ended the relationship*, 2 = *yes, but my partner and I are also dating other people*, 3 = *the relationship ended for some time but my partner and I are now back together*, and 4 = *yes, my partner and I are still exclusively involved in this relationship*. Based on a positive correlation between these items, *r* = 0.33, *p* = 0.001, they were converted to standard scores and averaged. Participants who would never forgive and were no longer in the relationship were considered unforgiving, whereas those who had forgiven and were still in the relationship were considered forgiving.

#### Procedure

Participants were recruited by approaching students in an advanced undergraduate psychology class and asking them to distribute a URL to the online study material. The students were to give the URLs to one male and one female non-student adult who had no relationship or association with each other. The participants had to be at least 18 years old, proficient in reading and writing English, able to use a computer and the internet, and must have currently, or previously, been in a romantic relationship. After signing the informed consent form, participants completed the demographic questions, information on their exercise behavior, transgression stimuli, and the forgiveness measure. The participants were debriefed in writing at the completion of data collection.

### Results

#### Main Analysis

**FIGURE 1:**
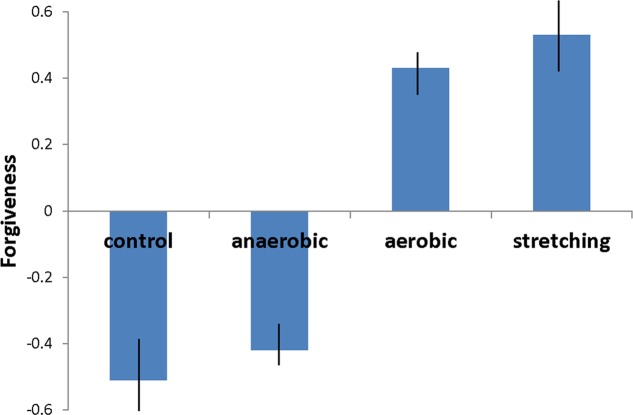
**The relations between exercise type and forgiveness (Study 1).** Significant (*p* < 05) differences were found between the stretching and control, stretching and anaerobic, aerobic and control, and aerobic and anaerobic conditions. Standardized scores and standard error bars.

A one-way between groups ANOVA was conducted to test the relation between routine exercise (none, anaerobic, aerobic, and stretching) and participants’ forgiveness of their romantic partner. A significant relation was found between the type of exercise participants routinely engaged in and forgiveness, *F*(3,88) = 7.31, *p* = 0.001, η^2^ = 0.21. Participants who engaged in aerobic exercise reported being significantly more forgiving, *M* = 0.43, *SD* = 1.01, 95% CI [0.11, 0.75], than those who did not exercise, *M* = -0.52, *SD* = 0.92, 95% CI [-1.03, 0.005], *t* = 3.44, *p* = 0.001, *d* = 0.75, and those who engaged in anaerobic exercise, *M* = -0.42, *SD* = 0.76, 95% CI [-0.74, 0.10], *t* = 3.65, *p* = 0.001, *d* = 0.56. Similarly, individuals who engaged in stretching exercises were significantly more forgiving, *M* = 0.53, *SD* = 0.75, 95% CI [-0.007, 1.07], than those who did not exercise, *M* = -0.52, *SD* = 0.92, *t* = 2.72, *p* = 0.008, *d* = 0.86, and those who engaged in anaerobic exercise, *M* = -0.42, *SD* = 0.76, *t* = 2.68, *p* = 0.009, *d* = 0.68 (see **Figure 1**). All of the effects were moderate to large according to Cohen (1988). No significant difference was found between the no exercise and anaerobic groups and the aerobic and stretching groups. As predicted, those who routinely engaged in stretching and aerobic exercises seemed to be more forgiving than non-exercising individuals and those who engaged in anaerobic exercises.

**FIGURE 2:**
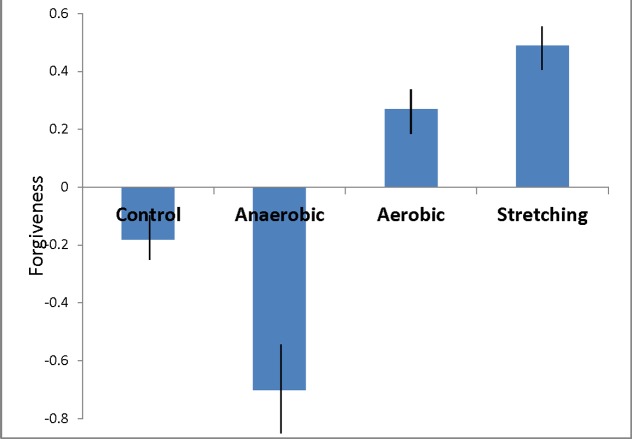
**The relations between exercise type and forgiveness (Study 2).** Standardized scores and standard error bars.

## Study 2

In Study 2 we created a real-time transgression in a field setting, manipulating and randomly assigning participants to the different exercise conditions to begin to test the causal relation between exercise and forgiveness. We used a single session of exercise for each modality incorporating moderately intense activities consistent with achieving an aerobic, anaerobic, and stretching effect. We also examined the extent to which the participant was able to override their grudges mediated the relation between type of exercise and forgiveness. The participants were told that they would be participating in an exercise class being conducted by a student enrolled in the Faculty of Kinesiology and that they would complete a brief Fitness Instructor Evaluation Form and a University Grade Form that would contribute to the student instructor’s actual grade in a course. Because of high demand on the exercise facilities on campus, we were limited to the same exercise studio for four 1 h sessions. In addition, the studio had a set capacity of 16 participants, and therefore, we were limited in how many participants we could test overall.

### Design

A one-way experimental design with exercise type as the Independent Variable (control, anaerobic, aerobic, and stretching) and forgiveness as the Dependent Variable was used.

### Method

#### Participants

Undergraduate students (*N* = 50, 25 women, 24 men, one did not report gender, *M*_age_ = 20.51, *SD* = 3.28) were recruited from the undergraduate research participant pool on campus. As described previously, our sample size was limited to a maximum of 16 participants per session because of the size of the workout studio. In total, 50 participants completed the study (control *n* = 13, anaerobic *n* = 11, aerobic *n* = 15, stretching *n* = 11). We ran a *post hoc* power analysis to determine the level of power we obtained for testing our mediation hypothesis. Consistent with Study 1, no differences were found between the control condition and the anaerobic training condition (i.e., our control conditions) nor between the anaerobic training and the flexibility training conditions (i.e., our treatment conditions) on the key dependent variables and therefore we combined them into 2 groups, (1) control + anaerobic training and (2) aerobic + flexibility training, to run this analysis. Based on a large effect size, *d* = 0.82, α = 0.05, and *N* = 50, our *post hoc* power for this analysis was 79%. The participants received credit toward their introductory psychology course grade in exchange for their participation. They reported exercising regularly (*M* = 4 times per week, *SD* = 1.50) and having a moderately high level of perceived fitness (*M* = 4.77, *SD* = 1.00, 7-point scale where 1 = *Not at all fit*, 7 = *Extremely fit*).

#### Materials

##### Exercise

We operationalized our exercise independent variable as four different exercise classes that trained moderately intense physical activity consistent with achieving aerobic, anaerobic, and stretching effects: control (one 30 min nutrition class), anaerobic training (one 30 min weight lifting class with 2 min sets and a 1 min rest between sets using weight machines), aerobic training (one 30 min aerobics class with continuous and sustained exercise throughout the session), and flexibility training (one 30 min stretching class with continuous stretching throughout the session, stretches were held for 30 s each on a floor mat). A senior kinesiology undergraduate student who was also a trained fitness instructor in anaerobic, aerobic, and stretching techniques served as our transgressor and fitness instructor. Based on her education, training, and applied fitness experience, the instructor played a significant role in designing and pilot testing our exercise conditions. The instructor was not informed about the focus on forgiveness or about the hypotheses of the study.

##### Transgression stimulus

The instructor showed up 12 min late for each session.

##### Transgression measures

Participants were asked to indicate the extent to which the instructor arrived on time, 1 = *Not at all*, 7 = *Very much so*, and the impact of the event on them, 1 = *Extremely negative*, 7 = *Extremely positive*.

##### Perceived physical functioning

Physical fitness is defined as a set of attributes that individuals achieve such as cardiorespiratory endurance and muscle strength (Caspersen et al., 1985). Based on this definition we operationalized perceived physical functioning as the extent to which the participants perceived themselves to be physically fit, energetic, active, and strong. Participants responded to each item using a 7-point scale ranging from 1 *Not at all* to 7 *Very much so*.

Our pilot testing has shown that participants who are expecting to participate in a study regarding the evaluation and grade of another become suspicious of the actual nature of the study when presented with a questionnaire that explicitly measures their forgiveness following a supposed unforeseen transgression. To minimize participants’ suspicion about the nature of the study and possible demand characteristics, we developed a procedure and set of non-explicit measures that conformed to the conceptual definition of our constructs and made sense to the participants regarding the circumstances of the study. Based on our previous research, in which a seemingly unexpected transgression occurred (Struthers et al., 2008), we had the researcher mention that in such situations, the participants were required to complete a “Disrupted Session Form” regarding their experience. On this form, participants described what happened and responded to items concerning the negative event, the impact it had on them, how much they thought about it during the session (rumination), and the extent to which they thought they could let go of the event.

In addition, forgiveness has been conceptually defined as a change in evaluative judgments and acts toward a transgressor from unfavorable/retaliatory to more favorable/beneficial (McCullough, 2001), and therefore, we included items that measured evaluative judgments toward the transgressor and a University Grading Form that measured participants’ grades for the transgressor (Worthington et al., 2015).

##### Grudge

Previous research has shown that rumination about (persistent thinking), and not letting go of, a transgression are related to self-control and grudges and therefore we measured participants’ rumination about the negative event and the extent to which they had difficulty letting go of the negative event (Denson et al., 2011; Burnette et al., 2014). We used the following items: First, we assessed whether participants ruminated about the instructor showing up late for the session, i.e., “How often were you thinking about the event during the session?”, and second, we assessed the extent to which the event was perceived as difficult for them to let go of, i.e., “To what extent will the event be difficult to let go of?”. Participants responded to the items on a 7-point scale ranging from 1 *Not at all* to 7 *Very much so*. We reasoned that the more participants thought about the event during the session (i.e., rumination) and the more they felt that it was difficult to let go of (i.e., holding a grudge), the less capacity they had to exert self-control over their grudges. This is based on previous theorizing that rumination and holding a grudge following transgressions leads to deficits in self-control (Denson et al., 2011; Burnette et al., 2014). Research shows that these measures produce similar associations following self-control tasks (Hagger et al., 2010).

##### Forgiveness

Consistent with definitions of forgiveness in which victims transform unfavorable evaluations and behaviors into more favorable evaluations and behaviors that benefit the transgressor (see Exline and Baumeister, 2000; McCullough, 2001; Struthers et al., 2008; Carlisle et al., 2012; Dorn et al., 2013; Worthington et al., 2015), we assessed forgiveness using a self-reported evaluation of the instructor and a university grading form. For the self-reported evaluation, participants evaluated how well the instructor interacted with others in the class, how motivating she was, and how satisfied they were with her. A 7-point scale, 1 = *Not at all*, 7 = *Very much so*, was used to measure all items. Participants were also given a formal grading form used by the University Faculty and they were instructed to assign a grade to the instructor that would contribute to her actual course grade. Participants were instructed to grade the instructor’s performance by choosing a letter grade that ranged from *F* to *A*+. Forgiveness was operationalized as more favorable self-reported evaluations of the instructor and higher assigned grades.

#### Procedure

Participants were randomly assigned to one of the four conditions. Participants were instructed to meet a research assistant at the entrance of the campus fitness center. Sessions were run in groups of 11 to 15 participants. Once all scheduled participants had arrived, they were led into a workout studio. Next they were given a brief summary of the study and an informed consent form. The participants were told that the purpose of the study was to assess new fitness instructors being trained within the Faculty of Kinesiology. Each participant read and signed the informed consent agreement and completed a demographic and exercise behavior questionnaire. To rig the transgression, the instructor arrived 12 min late for each session. While the participants were waiting, the research assistant checked her watch, looked outside the door, and said twice during the 12 min late period “The instructor is late. When she arrives we will start.” When the instructor arrived she immediately introduced herself and did not apologize or mention her tardiness. Next, the participants were instructed to begin their class. In the anaerobic training condition the participants were led by the instructor through a 30 min circuit of weight machines with 2 min allotted for each set and a 1 min rest between sets. A 5 min cool down occurred at the end of the exercise session. In the aerobic training condition, the instructor led them through a 30 min aerobic workout with continuous and sustained exercise including a 5 min cool down at the end of the session. In the stretching condition the participants were led through a 30 min yoga session and a 5 min cool down at the end. In the control condition the instructor led a 30 min lecture about sugary foods and drinks, the effects of these foods on the body, and healthier alternatives. At the end of each session, the instructor thanked the students and left the room. The Disruption in Session Form, Fitness Instructor Evaluation Form, and the University Grade Form were distributed to the participants by the research assistant. All sessions were completed in less than 1 h and the participants were debriefed in writing at the end of the data collection period.

### Results

#### Composite Variables and Manipulations

Based on positive inter-item correlations, items from the respective scales were averaged: perceived physical functioning, *M* = 4.99, *SD* = 1.27, α = 0.87; grudge, *M* = 2.47, *SD* = 1.32, *r* = 0.22; self-reported forgiveness, *M* = 5.49, *SD* = 1.16, α = 0.86. As well, a positive correlation was found between the self-reported forgiveness scale and assigned grade (*M* = 3.52, *SD* = 1.78), *r* = 0.48. Overall participants perceived that the instructor was late, *M* = 1.84, *SD* = 1.33, and reported that it had a moderately negative impact on them, *M* = 3.60, *SD* = 1.27. As desired, responses regarding the transgression did not vary as a function of the type of exercise, *F*s < 1.0.

A significant effect was found for type of exercise on the measure of perceived physical functioning, *F*(3, 49) = 2.82, *p* < 0.05, η^2^ = 0.16. We expected the participants who were in the exercise conditions to report greater perceived physical functioning after the training than the control group. Although the means were all in the expected direction, participants in the aerobic training condition reported significantly higher perceived physical functioning, *M* = 5.69, *SD* = 0.87, than the stretching condition, *M* = 4.64, *SD* = 1.02, *d* = 1.10, and the control condition, *M* = 4.51, *SD* = 1.51, *d* = 0.96, critical *t* = 2.02, *MSE* = 1.44, *p* < 0.05. No other significant differences were found.

#### Mediation Analysis

We used Hayes’ (2012) process and bootstrapping procedure to test the mediating role of grudges in explaining the relation between exercise and our self-reported and grade forgiveness scales.^1^ No statistically significant differences were found between the control and anaerobic training groups (i.e., control conditions) and between the aerobic training and stretching groups (i.e., treatment conditions) on the forgiveness variables (all *p*s < 0.05), and therefore, we dummy coded our exercise independent variables before testing our mediation predictions such that 0 = control + anaerobic training and 1 = aerobic training + stretching. For the self-reported forgiveness measure, a significant direct effect was found for exercise type, β = 0.63, *t* = 2.14, *p* = 0.038, 95% CI [0.04, 1.22]. As predicted, participants in the aerobic and stretching condition were more forgiving than participants in the control and anaerobic condition. In addition, the indirect effect of grudge was significant, β = 0.23, *SE* = 0.17, 95% CI [0.01, 0.81], suggesting that the relation between exercise type and forgiveness is mediated by the self-regulation of grudges. Participants in the aerobic and stretching condition were more likely to override their grudges, which in turn led to greater forgiveness.

For the grade forgiveness measure, a significant direct effect was also found for exercise type, β = 1.14, *t* = 2.76, *p* = 0.008, 95% CI [0.31, 1.96]. Participants in the aerobic and stretching condition awarded higher grades to the transgressor than did those in the anaerobic and control condition. However, grudge did not mediate the relation between exercise type and grade, β = 0.004, *SE* = 0.16, 95% CI [-0.27, 0.38].

## General Discussion

This research explored the idea that individuals who engage in certain types of exercise might improve relationships following a transgression by overriding grudges and forgiving. The results from two exploratory studies suggest that those who engage in aerobic and flexibility (i.e., stretching) exercises are more forgiving following a transgression than those who engage in anaerobic exercise. These findings also suggest that self-control of grudges mediates the relation between exercise and self-reported forgiveness for those in the aerobic and flexibility conditions but not between exercise and assigning course grades. The findings with respect to the association between exercise type and forgiveness in our non-experimental retrospective study (Study 1) were replicated in our field experiment (Study 2).

Moreover, in Study 2 we measured grudges to test their mediational role in explaining the relation between exercise and forgiveness. Our mediation results showed that overriding grudges only mediated the relation between exercise and self-reported forgiveness for those in the anaerobic and stretching conditions. One possible explanation for the null finding on grading was that our self-reported forgiveness measure and grading measure were unrelated; however, this was not the case as they had a reasonably strong positive relation. Perhaps exercise relates to intrapsychic forgiveness by influencing victims’ emotion regulation, which in turn, influences victims’ intrapsychic evaluation of the transgressor. Another explanation is that a victim’s intrapsychic forgiveness might take place before forgiving acts such as prosocial behavior. Future research examining this temporal sequence would be an asset to the forgiveness literature.

Although exploratory, we believe that this research has a number of positive aspects including systematically replicable results that generalize to different samples and research methods. An additional positive aspect of this research is the generalizability of the benefit of exercise from physical and psychological domains to social domains. These results suggest that training in stretching and aerobics exercises might play a meaningful role in sustaining relationships through the facilitation of forgiveness following an interpersonal transgression. However, it is also important to note a number of limitations of this research.

Because of limited resources associated with Study 2, such as access to exercise facilities, we had to make a number of methodological tradeoffs including a lower overall N than desired and more robust procedures and measures for demonstrating aerobic and anaerobic effects. As a result, our findings should be interpreted as exploratory. Future research that focuses on improving our methodological limitations such as increased statistical power and physiological measures to establish aerobic and anaerobic effects would enable directional hypotheses, stronger tests of the hypotheses, and stronger conclusions. Another limitation of this research was its focus on forgiveness as the only post-transgression response. There are other common post-transgression reactions that affect social bonds, such as seeking revenge (McCullough, 2008; McCullough et al., 2013). We believe that future research should include vengeance in order to explore the link between those who engage in specific types of exercise and getting back at someone. Revenge responses might be more relevant to certain types of exercise, such as anaerobic. For instance, exercise that does not promote physical functioning and increases testosterone levels such as lifting weights or circuit training, might promote a physical readiness to retaliate. Although our research tested the role of self-control in overriding grudges as an explanation for why exercise affects forgiveness, it did not test how mode of exercise influences self-control. A number of possibilities for future research have been identified in the exercise and self-control literature such as use of glucose, allocation of glucose via cerebral blood flow, shift in motivation, and self-regulatory fatigue (Beedie and Lane, 2012; Baumeister and Vohs, 2016; Beurms and Miller, 2016; Evans et al., 2016).

Overall, these studies suggest that forgiveness might be facilitated by aerobic and stretching exercises suggesting that exercise can play an important role in the repair of relationships following a transgression. Although the growth of gyms, aerobics studios, and yoga studios is encouraging, the activity levels of North Americans have generally been declining. This research provides yet another reason, arguably one of the most important, to get off the couch: improving relationships.

## Ethics Statement

York University Human Participant Research Committee (HPRC). All participants were informed of the nature of the study and what they would be doing. Data collection proceeded after the participants signed the informed consent form. No vulnerable populations were involved in this research.

## Author Contributions

All authors listed, have made substantial, direct and intellectual contribution to the work, and approved it for publication.

## Conflict of Interest Statement

The authors declare that the research was conducted in the absence of any commercial or financial relationships that could be construed as a potential conflict of interest.

## Footnotes

1 A one-way between groups ANOVA was conducted to examine the effect of type of exercise training on our self-reported forgiveness and grade measures. Because they were correlated, *r* = 0.48, and the patterns of results were similar for both measures, to simplify our analysis, we decided to combine them into one analysis by converting them to standardized scores and averaging them. A significant effect was found for forgiveness, *F*(3,48) = 3.45, *p* = 0.02, η^2^ = 0.19. As predicted, significant differences were found between the aerobic exercise, *M* = 0.27, *SD* = 0.98, 95% CI [-0.27, 0.81], and anaerobic exercise conditions, *M* = -0.70, *SD* = 1.01, *d* = 1.05, 95% CI [-1.38, 0.15], *t* = 2.54, *p* = 0.01 (see **Figure 2**). As well, a significant difference and relatively strong effect was found between the stretching, *M* = 0.49, *SD* = 0.63, 95% CI [0.03, 0.95], and anaerobic exercise conditions, *d* = 1.35, *t* = 2.91, *p* < 0.01. A marginally non-significant difference was found between the stretching and control conditions, *M* = -0.18, *SD* = 1.02, 95% CI [-0.80, 0.44], *d* = 0.91, *t* = 1.76, *p* = 0.08. All of these effects are relatively strong according to Cohen (1988). No significant differences were found between aerobic and stretching exercise.

## References

[B1] Antúnez J. M. Navarro J. F. Adan A. (2013). Circadian typology and emotional intelligence in healthy adults. *Chronobiol. Int.* 30 981–987. 10.3109/07420528.2013.790397 23834706

[B2] Aquino K. Douglas S. (2003). Identity threat and antisocial behaviour in organizations: the moderating effects of individual differences, aggressive modeling, and hierarchical status. *Organ. Behav. Hum. Decis. Process.* 90 195–208. 10.1016/S0749-5978(02)00517-4

[B3] Audiffren M. Andre N. (2015). The strength model of self-control revisited: linking acute and chronic effects of exercise on executive functions. *J. Sport Health Sci.* 4 30–46. 10.1016/j.jshs.2014.09.002

[B4] Barnes J. N. (2015). Exercise, cognitive functioning, and aging. *Adv. Physiol. Educ.* 39 55–62. 10.1152/advan.00101.2014 26031719 PMC4587595

[B5] Batty D. Thune I. (2000). Does physical activity prevent cancer? *Br. Med. J.* 321 1424–1425. 10.1136/bmj.321.7274.1424 11110720 PMC1119154

[B6] Baumeister R. F. (2014). Self-regulation, ego depletion, and inhibition. *Neuropsychologia* 65 313–319. 10.1016/j.neuropsychologia.2014.08.012 25149821

[B7] Baumeister R. F. DeWall C. N. Ciarocco N. J. Twenge J. M. (2005). Social exclusion impairs self-regulation. *J. Pers. Soc. Psychol.* 88 589–604. 10.1037/0022-3514.88.4.589 15796662

[B8] Baumeister R. F. Exline J. J. Sommer K. L. (1998). “The victim role, grudge theory, and two dimensions of forgiveness,” in *Dimensions of Forgiveness: Psychological Research and Theological Perspective*, ed. Worthington E. L. Jr. (Philadelphia, PA: Templeton Foundation Press).

[B9] Baumeister R. F. Leary M. R. (1995). The need to belong: desire for interpersonal attachments as a fundamental human motivation. *Psychol. Bull.* 117 497–529. 10.1037/0033-2909.117.3.497 7777651

[B10] Baumeister R. F. Vohs K. D. (2016). “Strength model of self-regulation as a limited resource: assessment, controversies, update,” in *Advances in Experimental Social Psychology* Vol. 54 eds Olsen J. Zanna M. (Amsterdam: Elsevier Inc), 67–127. 10.1016/bs.aesp.2016.04.001

[B11] Beedie C. J. Lane A. M. (2012). The role of glucose in self-control another look at the evidence and an alternative conceptualization. *Pers. Soc. Psychol. Rev.* 16 143–153. 10.1177/1088868311419817 21896791

[B12] Berscheid E. Reis H. T. (1998). “Attraction and close relationships,” in *Handbook of Social Psychology*, 4th Edn Vol. 2 eds Gilbert D. T. Fiske S. T. Lindsey G. (New York, NY: McGraw-Hill), 193–281.

[B13] Beurms S. Miller H. C. (2016). Sharing more than the sofa: what dogs can teach us about human self-control. *Psychol. Sci.* 25 351–356. 10.1177/0963721416664392

[B14] Bowlby J. (1969). *Attachment and Loss: Attachment*, Vol. 1 New York, NY: Basic Books

[B15] Braithwaite S. R. Selby E. A. Fincham F. D. (2011). Forgiveness and relationship satisfaction: mediating mechanisms. *J. Fam. Psychol.* 25 551–559. 10.1037/a0024526 21707170 PMC3156929

[B16] Budde H. Boelcker-Rehage C. PietraByk-Kendziorra S. Ribeiro P. Tidow G. (2008). Acute coordinative exercise improves attentional performance in adolescents. *Neurosci. Lett.* 441 219–223. 10.1016/j.neulet.2008.06.024 18602754

[B17] Burnette J. L. Davisson E. K. Finkel E. J. Van Tongeren D. R. Hui C. M. Hoyle R. H. (2014). Self-control and forgiveness: a meta-analytic review. *Soc. Psychol. Pers. Sci.* 5 443–450. 10.1177/1948550613502991

[B18] Buss D. M. (1990). The evolution of anxiety and social exclusion. *J. Soc. Clin. Psychol.* 9 196–210. 10.1521/jscp.1990.9.2.196

[B19] Carlisle R. D. Tsang J. Ahmad N. Y. Worthington E. L. Jr. Witvliet C. V. Wade N. (2012). Do actions speak louder than words? Differential effects of apology and restitution on behavioral and self-report measures of forgiveness. *J. Posit. Psychol.* 7 294–305. 10.1080/17439760.2012.690444

[B20] Carter E. C. McCullough M. E. (2014). Publication bias and the limited strength model of self-control: has the evidence for ego depletion been overestimated? *Front. Psychol.* 5:823 10.3389/fpsyg.2014.00823 PMC4115664 25126083

[B21] Caspersen C. J. Powell K. E. Christenson G. M. (1985). Physical activity, exercise, and physical fitness: definitions and distinctions for health-related research. *Public Health Rep.* 100 126–131. 3920711 PMC1424733

[B22] Castro C. M. Wilcox S. O’Sullivan P. Bauman K. King A. C. (2002). An exercise program for women who are caring for relatives with dementia. *Psychosom. Med.* 64 458–468. 10.1097/00006842-200205000-00010 12021419

[B23] Chang Y. K. Etnier J. L. (2015). Acute exercise and cognitive function: emerging research issues. *J. Sport Health Sci.* 4 1–3. 10.1016/j.jshs.2014.12.001 PMC6620473 31333882

[B24] Chang Y. K. Ku P. W. Tomporowski P. D. Chen F. T. Huang C. C. (2012a). The effects of acute resistance exercise on late-middle-aged adults’ goal planning. *Med. Sci. Sports Exerc.* 44 1773–1779. 10.1249/MSS.0b013e3182574e0b 22460477

[B25] Chang Y. K. Labban J. D. Gapin J. I. Etnier J. L. (2012b). The effect of acute exercise on cognitive performance: a meta-analysis. *Brain Res.* 102 421–428. 10.1016/j.brainres.2012.02.068 22480735

[B26] Cohen J. (1988). *Statistical Power Analysis for the Behavioral Sciences*, 2nd Edn Mahwah, NJ: Lawrence Erlbaum Associates.

[B27] Colcombe S. Kramer A. F. (2003). Fitness effects on the cognitive function of older adults: a meta-analytic study. *Psychol. Sci.* 14 125–130. 10.1111/1467-9280.t01-1-01430 12661673

[B28] Corsica J. A. Perry M. G. (2003). “Obesity,” in *Handbook of Psychology: Health Psychology* Vol. 9 eds Nezu A. M. Nezu C. M. Geller P. A. (New York, NY: Wiley).

[B29] Cotman C. W. Berchtold N. C. Christie L. A. (2007). Exercise builds brain health: key roles of growth factor cascades and inflammation. *Trends Neurosci.* 30 464–472. 10.1016/j.tins.2007.06.011 17765329

[B30] Cunningham M. R. Baumeister R. F. (2016). How to make nothing out of something: analyses of the impact of study sampling and statistical interpretation in misleading meta-analytic conclusions. *Front. Psychol.* 7:1639 10.3389/fpsyg.2016.01639 PMC5079083 27826272

[B31] Davranche K. McMorris T. (2009). Specific effects of acute moderate exercise on cognitive control. *Brain Cogn.* 69 565–570. 10.1016/j.bandc.2008.12.001 19138814

[B32] Denson T. F. Pederson W. C. Friese M. Hahm A. Roberts L. (2011). Understanding impulsive aggression: angry rumination and reduced self-control capacity are mechanisms underlying the provocation-aggression relationship. *Pers. Soc. Psychol. Bull.* 37 850–862. 10.1177/0146167211401420 21421767

[B33] DeWall C. N. Pond R. S. Bushman B. J. (2010). Sweet revenge: diabetic symptoms predict less forgiveness. *Pers. Individ. Differ.* 49 823–826. 10.1016/j.paid.2010.06.030

[B34] Diamond A. (2006). “The early development of executive functions,” in *Lifespan Cognition: Mechanisms of Change*, eds Bialystok E. Craik F. I. M. (New York, NY: Oxford University Press), 70–95. 10.1093/acprof:oso/9780195169539.003.0006

[B35] Diamond A. Barnett W. S. Thomas J. Munro S. (2007). Preschool program improves cognitive control. *Science* 318 1387–1388. 10.1126/science.1151148 18048670 PMC2174918

[B36] Dorn K. Hook J. N. Davis D. E. Van Tongeren D. R. Worthington E. L. Jr. (2013). Behavioral methods of assessing forgiveness. *J. Posit. Psychol.* 9 75–80. 10.1080/17439760.2013.844267

[B37] Dunn A. L. Trivedi M. H. O’Neal H. A. (2001). Physical activity dose-response effects on outcomes of depression and anxiety. *Med. Sci. Sports Exerc.* 33 S587–S597. 10.1097/00005768-200106001-00027 11427783

[B38] Eaton J. Struthers C. W. (2006). The reduction of psychological aggression across varied interpersonal contexts through repentance and forgiveness. *Aggress. Behav.* 32 195–206. 10.1002/ab.20119

[B39] Erickson K. I. Leckie R. L. Weinstein A. M. (2014). Physical activity fitness, and gray matter volume. *Neurobiol. Aging* 35 520–528. 10.1016/j.neurobiolaging.2014.03.034 24952993 PMC4094356

[B40] Erickson K. I. Voss M. W. Prakash R. S. Basak C. Szabo A. Chaddock L. (2015). Exercise training increases size of hippocampus and improves memory. *Proc. Natl. Acad. Sci. U.S.A.* 108 3017–3022. 10.1073/pnas.1015950108 PMC3041121 21282661

[B41] Evans D. R. Boggero I. A. Segerstrom S. C. (2016). The nature of self-regulatory fatigue and “ego depletion”: lessons from physical fatigue. *Pers. Soc. Psychol. Rev.* 20 291–310. 10.1177/1088868315597841 PMC4788579 26228914

[B42] Exline J. J. Baumeister R. F. (2000). “Expressing forgiveness and repentance: benefits and barriers,” in *The Psychology of Forgiveness*, eds McCullough M. E. Pargament K. I. Thoresen C. E. (New York, NY: Guilford), 133–155.

[B43] Exline J. J. Baumeister R. F. Bushman B. J. Campbell W. K. Finkel E. J. (2004). Too proud to let go: narcissistic entitlement as a barrier to forgiveness. *J. Pers. Soc. Psychol.* 87 894–912. 10.1037/0022-3514.87.6.894 15598113

[B44] Fincham F. D. Beach S. R. H. Davila J. (2004). Forgiveness and conflict resolution in marriage. *J. Fam. Psychol.* 18 72–81. 10.1037/0893-3200.18.1.72 14992611

[B45] Finkel E. J. Campbell W. K. (2001). Self-control and accommodation in close relationships: an interdependence analysis. *J. Pers. Soc. Psychol.* 81 263–277. 10.1037/0022-3514.81.2.263 11519931

[B46] Friedenreich C. M. Bryant H. E. Courneya K. S. (2001). Case-control study of lifetime physical activity and breast cancer risk. *Am. J. Epidemiol.* 154 336–347. 10.1093/aje/154.4.336 11495857

[B47] Gilliland F. D. Li Y. F. Baumartner K. Crumley D. Samet G. M. (2001). Physical activity and breast cancer risk in Hispanic and non-Hispanic white women. *Am. J. Epidemiol.* 154 442–450. 10.1093/aje/154.5.442 11532786

[B48] Gomez-Pinilla F. Hillman C. (2013). The influence of exercise on cognitive abilities. *Compr. Physiol.* 3 401–428. 10.1002/cphy.c110063 PMC3951958 23720292

[B49] Guiney H. L. Machado L. (2013). Benefits of regular aerobic exercise for executive functioning in healthy populations. *Psychon. Bull. Rev.* 20 73–86. 10.3758/s13423-012-0345-4 23229442

[B50] Hagger M. S. Wood C. Stiff C. Chatzisarantis N. L. D. (2010). Ego depletion and the strength model of self-control: a meta-analysis. *Psychol. Bull.* 136 495–525. 10.1037/a0019486 20565167

[B51] Harveson A. T. Hannon J. C. Brusseau T. A. Podlog L. Papadopoulos C. Durrant L. H. (2016). Acute effects of 30 minute resistance and aerobic exercise on cognition in a high school sample. *Res. Q. Exerc. Sport* 87 214–220. 10.1080/02701367.2016.1146943 26958898

[B52] Hayes A. F. (2012). *PROCESS: A Versatile Computational Tool for Observed Variable Mediation, Moderation, and Conditional Process Modeling.* Available at: http://www.afhayes.com/public/process2012.pdf

[B53] Heider F. (1958). *The Psychology of Interpersonal Relations.* New York, NY: Wiley 10.1037/10628-000

[B54] Hillman C. H. Erickson K. I. Kramer A. F. (2008). Be smart, exercise your heart: exercise effects on brain and cognition. *Nat. Rev. Neurosci.* 9 58–65. 10.1038/nrn2298 18094706

[B55] Hoyt W. T. Fincham F. D. McCullough M. E. Maio G. Davila J. (2005). Responses to interpersonal transgressions in families: forgivingness, forgivability, and relationship specific effects. *J. Pers. Soc. Psychol.* 89 375–394. 10.1037/0022-3514.89.3.375 16248720

[B56] Hung T. M. Tsai C. L. Chen F. T. Wang C. C. Chang Y. K. (2013). The immediate and sustained effects of acute exercise on planning aspects of executive function. *Psychol. Sport Exerc.* 14 728–736. 10.1016/j.psychsport.2013.05.004

[B57] Inzlicht M. Schmeichel B. J. Macrae C. N. (2014). Why self-control seems (but may not be) limited. *Trends Cogn. Sci.* 18 127–133. 10.1016/j.tics.2013.12.009 24439530

[B58] Johnson J. (1998). *Under the Microscope: Breathing, How We Use Air.* Danbury, CT: Grolier Educational, Sherman Turnpike.

[B59] Jung J. Kang H. Shim S. Cho K. Yu J. (2012). Effects of resistive exercise on cerebral blood flow velocity and pulsatility index of healthy people. *J. Phys. Ther. Sci.* 24 915–917. 10.1589/jpts.24.915

[B60] Karremans J. C. Van Lange P. A. M. (2004). Back to caring after being hurt: the role of forgiveness. *Eur. J. Soc. Psychol.* 34 207–227. 10.1002/ejsp.192

[B61] Karremans J. C. Van Lange P. A. M. Holland R. W. (2005). Forgiveness and its associations with prosocial thinking, feeling, and doing beyond the relationship with the offender. *Pers. Soc. Psychol. Bull.* 31 1315–1326. 10.1177/0146167205274892 16143664

[B62] Kato T. (2016). Effects of partner forgiveness on romantic break-ups in dating relationships: a longitudinal study. *Pers. Individ. Differ.* 95 185–189. 10.1016/j.paid.2016.02.050

[B63] Kokkinos P. Myers J. Kokkinos J. P. Pittaras A. Narayan P. Manolis A. (2007). Exercise capacity and mortality in black and white men. *Circulation* 117 614–622. 10.1161/CIRCULATIONAHA.107.734764 18212278

[B64] Larson E. B. Wang L. Bowen J. D. McCormick W. C. Teri L. Crane P. (2006). Exercise is associated with reduced risk for incident dementia among persons 65 years of age and older. *Ann. Intern. Med.* 144 73–81. 10.7326/0003-4819-144-2-200601170-00004 16418406

[B65] Lefferts W. K. Augustine J. A. Heffernan K. S. (2014). Effects of acute resistance exercise on carotid artery stiffness and cerebral blood flow pulsatility. *Front. Physiol.* 5: 1–10. 10.3389/fphys.2014.00101 24678301 PMC3958641

[B66] Levchuck C. M. Drohan M. Kosek J. K. (2000). *Healthy Living.* Detroit, MI: Gale Group.

[B67] Luchies L. B. Finkel E. J. McNulty J. K. Kumashiro M. (2010). The doormat effect: when forgiving erodes self-respect and self-concept clarity. *J. Pers. Soc. Psychol.* 98 734–749. 10.1037/a0017838 20438221

[B68] Mazzeo R. S. Cavanagh P. Evans W. J. Fiatarone M. Hagberg J. McAuley E. (1998). ACSM position stand: exercise and physical activity for older adults. *Med. Sci. Sports Exerc.* 30 992–1008. 10.1249/00005768-199806000-00033 9624662

[B69] McAtee R. E. Charland J. (2007). *Facilitated Stretching: PNF Stretching and Stretching Made Easy*, 3rd Edn Champaign, IL: Human Kinetics.

[B70] McCullough M. E. (2001). Forgiveness: who does it and how do they do it? *Curr. Dir. Psychol. Sci.* 10 194–197. 10.1111/1467-8721.00147

[B71] McCullough M. E. (2008). *Beyond Revenge: The Evolution of the Forgiveness Instinct.* San Francisco, CA: Jossey-Bass.

[B72] McCullough M. E. Bono G. Root L. M. (2007). Rumination, emotion, and forgiveness: three longitudinal studies. *J. Pers. Soc. Psychol.* 92 490–505. 10.1037/0022-3514.92.3.490 17352605

[B73] McCullough M. E. Kurzban R. Tabak B. A. (2013). Cognitive systems for revenge and forgiveness. *Behav. Brain Sci.* 36 1–58. 10.1017/S0140525X11002160 23211191

[B74] McCullough M. E. Rachal K. C. Sandage S. J. Worthington E. L. J. Brown S. W. Hight T. L. (1998). Interpersonal forgiving in close relationships: II. Theoretical elaboration and measurement. *J. Pers. Soc. Psychol.* 75 1586–1603. 10.1037/0022-3514.75.6.1586 9914668

[B75] Myers D. G. (2000). The funds, friends, and faith of happy people. *Am. Psychol.* 55 56–67. 10.1037///0003-066x.55.1.56 11392866

[B76] Oaten M. Cheng K. (2006). Longitudinal gains in self-regulation from regular exercise. *Br. J. Health Psychol.* 11 717–733. 10.1348/135910706x96481 17032494

[B77] Paillard T. (2015). Preventive effects of regular physical exercise against cognitive decline and the risk of dementia with age advancement. *Sports Med. Open* 1 1–6. 10.1186/s40798-015-0016-x PMC5005681 26284161

[B78] Paleari F. G. Regalia C. Fincham F. (2005). Marital quality, forgiveness, empathy, and rumination: a longitudinal analysis. *Pers. Soc. Psychol. Bull.* 31 368–378. 10.1177/0146167204271597 15657452

[B79] Pontifex M. B. Raine L. B. Johnson C. R. Chaddock L. Voss M. W. Cohen N. J. (2011). Cardiorespiratory fitness and the flexible modulation of cognitive control in preadolescent children. *J. Cogn. Neurosci.* 23 1332–1345. 10.1162/jocn.2010.21528 20521857

[B80] Rapske D. L. Boon S. D. Alibhai A. M. Kheong M. J. (2010). Not forgiven, notforgotten: an investigation of unforgiven interpersonal offenses. *J. Soc. Clin. Psychol.* 29 1100–1130. 10.1521/jscp.2010.29.10.1100

[B81] Ray R. D. Wilhelm F. H. Gross J. J. (2008). All in the mind’s eye? Anger rumination and reappraisal. *J. Pers. Soc. Psychol.* 94 133–145. 10.1037/0022-3514.94.1.133 18179323

[B82] Robinson M. D. Schmeichel B. J. Inzlicht M. (2010). A cognitive control perspective on self-control strength and its depletion. *Soc. Pers. Psychol. Compass* 4 199–200. 10.1111/j.1751-9004.2009.00244.x

[B83] Rusting C. L. Nolen-Hoeksema S. (1998). Regulating responses to anger: effects of rumination and distraction on angry mood. *J. Pers. Soc. Psychol.* 74 790–803. 10.1037/0022-3514.74.3.790 9523420

[B84] Ryan R. M. Deci E. L. (2000). Self-determination theory and the facilitation of intrinsic motivation, social development, and well-being. *Am. Psychol.* 55 68–78. 10.1037/0003-066X.55.1.68 11392867

[B85] Rye M. S. Pargament K. I. (2002). Forgiveness and romantic relationships in college: can it heal the wounded heart? *J. Clin. Psychol.* 58 419–441. 10.1002/jclp.1153 11920695

[B86] Scheinberg P. Blackburn L. I. Rich M. Saslaw M. (1954). Effects of vigorous physical exercise on cerebral circulation and metabolism. *Am. J. Medicine* 16 549–554. 10.1016/0002-9343(54)90371-X 13148198

[B87] Schmidt K.-H. Beck R. Rivkin W. Diestel S. (2016). Self-control demands at work and psychological strain: the moderating role of physical fitness. *Int. J. Stress Manag.* 23 255–275. 10.1037/str0000012

[B88] Seifert T. Secher N. H. (2011). Sympathetic influence on cerebral blood flow and metabolism during exercise in humans. *Progr. Neurobiol.* 95 406–426. 10.1016/j.pneurobio.2011.09.008 21963551

[B89] Singh N. A. Clements K. M. Singh M. A. F. (2001). The efficacy of exercise as a long-term antidepressant in elderly subjects: a randomized controlled trial. *J. Gerontol. A* 56 497–505. 10.1093/gerona/56.8.M497 11487602

[B90] Smith C. D. (2006). A comparison of the effects of anaerobic and aerobic exercise on mood. *Diss. Abstr. Int.* 67 561.

[B91] Smith P. J. Blumenthal J. A. Hoffman B. M. Cooper H. Strauman T. A. Welsh-Bohmer K. (2010). Aerobic exercise and neurocognitive performance: a meta-analytic review of randomized controlled trials. *Psychosom. Med.* 72 239–252. 10.1097/PSY.0b013e3181d14633 20223924 PMC2897704

[B92] Sonstroem R. J. (1997). “Physical activity and self-esteem,” in *Physical Activity and Mental Health*, ed. Morgan W. P. (Washington, DC: Taylor & Francis), 127–143.

[B93] Struthers C. W. Eaton J. Mendoza R. Santelli A. G. Shirvani N. (2010). Interrelationship Among injured parties’ attributions of responsibility, appraisal of appropriateness to forgive the transgressor, forgiveness, and repentance. *J. Appl. Soc. Psychol.* 40 970–1002. 10.1111/j.1559-1816.2010.00607.x

[B94] Struthers C. W. Eaton J. Santelli A. G. Uchiyama M. Shirvani N. (2008). The effects of attributions of intent and apology on forgiveness: when saying sorry may not help the story. *J. Exp. Soc. Psychol.* 44 983–992. 10.1016/j.jesp.2008.02.006

[B95] Tangney J. P. Baumeister R. F. Boone A. L. (2004). High self-control predicts less pathology, better grades, and interpersonal success. *J. Pers.* 72 271–324. 10.1111/j.0022-3506.2004.00263.x 15016066

[B96] Taylor S. E. Sirois F. M. (2009). *Health Psychology: Canadian Edition.* Toronto, ON: McGraw-Hill Ryerson Limited.

[B97] Thomas S. N. Schroeder T. Secher N. H. Mitchell J. B. (1987). Cerebral blood flow during submaximal and maximal dynamic exercise in humans. *J. Appl. Physiol.* 67 744–748. 10.1152/jappl.1989.67.2.744 2507500

[B98] Tooby J. Cosmides L. Price M. (2006). Cognitive adaptations for n-person exchange: the evolutionary roots of organizational behavior. *Manage. Decis. Econ.* 27 103–129. 10.1002/mde.1287 PMC3693395 23814325

[B99] Van der Kooy K. Rookus M. A. Verloop J. Van Leeuwen F. E. Peterse J. (2001). Physical activity and breast cancer risk. *Am. J. Epidemiol.* 153 S110.

[B100] Voelcker-Rehage C. Niemann C. (2013). Structural and functional brain changes related to different types of physical exercise activity across the life-span. *Neurosci. Biobehav. Rev.* 37 2268–2295. 10.1016/j.neubiorev.2013.01.028 23399048

[B101] Voss M. W. Prakash R. S. Erickson I. K. I. Basak C. Chaddock L. Kim J. S. (2010). Plasticity of brain networks n a randomized intervention trial of exercise in older adults. *Front. Aging Neruosci.* 2:32 10.3389/fnagi.2010.00032 PMC2947936 20890449

[B102] Witvliet C. V. Ludwig T. E. Vander Laan K. L. (2001). Granting forgiveness or harboring grudges: implications for emotion, physiology, and health. *Psychol. Sci.* 12 117–123. 10.1111/1467-9280.00320 11340919

[B103] Worthington E. L. Jr. Lavelock C. R. Witvliet C. V. O. Rye M. S. Tsang J. Toussaint L. (2015). *Measures of Forgiveness. Measures of Personality and Social Psychological Constructs.* Chennai: Elsevier.

[B104] Ysseldyk R. Wohl M. J. A. (2012). I forgive therefore I’m committed: a longitudinal examination of commitment after a romantic relationship transgression. *Can. J. Behav. Sci.* 44 257–263. 10.1037/a0025463

[B105] Zheng G. Li S. Huang M. Liu F. Tao J. Chen L. (2015). The effect of Tai Chi training on cardiorespiratory fitness in healthy adults: a systematic review and meta-analysis. *PLoS ONE* 10:e0117360 10.1371/journal.pone.0117360 PMC4332633 25680184

[B106] Zoeller R. F. Jr. (2007). Physical activity and fitness in the prevention of coronary heart disease and associated risk factors. *Am. J. Lifestyle Med.* 1 29–33. 10.1177/1559827606293845

